# Can Genomics Remove Uncertainty from Adoption? Social Workers’ and Medical Advisors’ Accounts of Genetic Testing

**DOI:** 10.1093/bjsw/bcab017

**Published:** 2021-02-24

**Authors:** Michael Arribas-Ayllon, Katherine Shelton, Angus Clarke

**Affiliations:** 1 School of Social Sciences, Cardiff University, Cardiff, Wales, UK; 2 School of Psychology, Cardiff University, Cardiff, Wales, UK; 3 Medical Genetics, University of Wales College of Medicine, Cardiff, Wales, UK

**Keywords:** accounts, adoption, ethics, genetic testing, genomics, permanency, uncertainty

## Abstract

Genetic testing is controversial in adoption with professionals taking different positions on whether children should be protected from genetic information or whether it can be used to assist adoption. In this article, we argue that advances in ‘genome-wide’ testing add further complications to these debates. Although next-generation sequencing (NGS) and microarray-based technologies can offer high-quality molecular diagnoses for a variety of conditions, they also increase the burden of interpretation. For these reasons, adoption professionals will need to understand the relevance and complexity of biomedical information. Our study explores the accounts of social workers’ and medical advisors’ knowledge and reasoning about genetic testing in adoption. Twenty participants, including social workers, managers, medical advisors and paediatricians, were recruited from adoption services in England and Wales. A key finding revealed that medical professionals reported increasing pressure to test children prior to adoption, whilst social workers justified testing on the basis that it reduced uncertainty and therefore assisted adoption. Professionals’ accounts of genetic testing suggest that social workers may not be aware of the potential *indeterminacy* of microarray and NGS technologies. This has important implications for adoption because increases in genomic uncertainty can stigmatise children and disadvantage their prospects for adoption.

## Introduction

Genetic testing is controversial in adoption. Professionals take different positions on whether children should be protected from genetic information or whether it can be used to assist adoption. These positions stem from debates in the 1990s that now inform genetics policy in the UK. The consensus view is that decisions regarding the genetic testing of healthy children must respect the child’s autonomy. Despite these guidelines, anecdotal evidence suggests that medical professionals are frequently pressurised by adoption teams and prospective adopters to carry out testing to ‘guarantee’ the child’s future health. Often implied by such requests is the removal of medical uncertainty from decision-making around adoption.

The availability of ‘genome-wide’ testing adds further complications to these debates. We argue that previous ethical thinking based on predictive genetic testing assumes that a clear result would reduce uncertainty. However, new forms of diagnostic testing are likely to produce information that increases it. In this study, we explore the accounts of social workers and medical advisors discussing cases of genetic testing and their understanding of uncertainty in adoption. It is necessary to consider several factors to understand the context in which genetic information is used: we need to understand the changing profile of the adopted child, the recent policy on avoiding delays in adoption and the increasing acceptance of genetics as an explanatory ‘tool’ for health assessment. Together, these factors have shaped a system that has become less tolerant of uncertainty, especially when efforts are concentrated on securing permanence for looked after children.

## Changing profile of adoption

The profile of the adopted child today is very different from the classic model of adoption. Traditionally, adoption in the UK was a private process involving healthy white babies relinquished by birth parents to culturally similar adopters. Reflecting the values of the late Victorian era, ‘closed’ adoption served to reinforce the legitimacy of the marital family unit (O’Halloran, 2015). After the legalisation of abortion in 1968, adoptions consistently declined, with far fewer babies available and with adopted children more likely to be ethnically dissimilar from their adopters. Open adoption practices emerged from increasing recognition of the fluid and impermanent characteristics of modern families as well as the right of adoptees to access information about their medical and birth family history (Hill *et al.*, 2010).

Today, UK adoption agencies work to secure permanence for children who are the subject of care orders. Looking after children is substantially more likely to have Special Educational Needs (SEN) with 55.9 per cent requiring SEN support compared with 14.9 per cent of all children in England (Department for Education [DfE], 2019). Many of these children have complex physical and/or health-related problems and developmental delay, with diverse causes including genetic factors and foetal alcohol syndrome (DfE, 2015). Despite the diversity of contemporary adoptees, there is a persistent mismatch between the expectations of adopters and the characteristics of children available (Cousins, 2009). Adopters are less likely to consider children with learning disabilities or behavioural disorders, especially those whose problems are likely to have a genetic origin (Kingston, 2007).

In addition to meeting the challenges of placing children, adoption specialists also face institutional pressures to speed up adoption processes. Under Blair’s Labour government, the Adoption and Children Act (2002) sought to overturn the culture of adoption from serving middle-class interests in creating a system that supported the diverse needs of children (Bunt, 2014). A key strategy was to widen the pool of adopters to facilitate the placement of children with disabilities, many of whom were in long-term looked after care. The rise of ‘permanency planning’ (Fein and Maluccio, 1992) is another key policy that strives to secure a sense of security, continuity and identity for looked after children. Under this framework, local authorities may pursue adoption as the best option for a looked after child (DfE, 2011). Agencies are therefore under considerable pressure to facilitate a good match for children with diverse needs.

## Health assessments and genetic testing

It is important that prospective adopters are fully informed about the child’s early life experiences. Under the Adoption Act Regulations (2005), agencies have a legal duty to obtain and communicate health information, including the child’s birth family history. However, background information is often incomplete or unreliable (Hill and Edwards, 2009). Furthermore, social workers may not be equipped to communicate the technical aspects of disabilities or medical risks. Medical advisors play a key role in providing health information to prospective adopters. In conjunction with social workers, they conduct health assessments and collate information which is crucial in informing the adoption agency’s matching process (Sampeys and Barnes, 2017).

In recent decades, there has been increasing acceptance of genetics as an explanatory tool in adoption. During health assessment, a medical advisor may refer a child to clinical genetics for three main reasons: (i) the child presents with features suggestive of an underlying genetic disorder; (ii) the child is unaffected but family history raises concern about future risk and (iii) there is a history of substance misuse or infections during pregnancy, most commonly associated with foetal alcohol exposure. Clinical genetic assessment involves physical examination for features suggestive of an underlying disorder, as well as gathering background information about the child’s medical and family history. However, without the cooperation or consent of birth parents, genetic assessment will be limited (Hill *et al.*, 2010). Lingering diagnostic uncertainty may warrant further investigations, such as genetic testing, as part of working up a thorough clinical assessment (Parker *et al.*, 2016).

There are three broad categories of testing in medical genetics: *diagnostic testing* can target specific genes or scan the entire genome for variations to aid clinical diagnosis. The most common instances of diagnostic testing in adoption are cases of suspected developmental delay or foetal alcohol syndrome. *Predictive testing* applies to inherited conditions with an onset usually in adult life, as with Huntington’s disease, or later in childhood. Testing positive means the individual *will* develop the disorder, though age of onset is variable. *Carrier testing* is usually carried out on unaffected individuals at risk of transmitting recessive or sex-linked conditions to their own future children, such as cystic fibrosis or haemophilia (Turnpenny and Ellard, 2017).

In all these cases, a request to perform testing on a child requires consent from a parent or an individual with parental responsibility. However, if there is no immediate medical benefit to the child, then the recommendation is that testing should be deferred until the child is able to give consent. There are circumstances where genetic testing may be requested to explain developmental problems, despite the indications being too weak to justify such testing in another child. The fact that a child is looked after is sometimes used to lower the threshold for genetic investigations (Newson and Leonard, 2010).

## Ethics of genetic testing in adoption

Following the first wave of predictive tests developed in the 1980s and 1990s, policy debates considered whether genetic information was ‘exceptional’ and required its own special policies to protect individuals from discrimination. Similar arguments have been made about genetic testing of children. Predictive genetic testing for Huntington’s disease became a model for recommending that children should not be tested unless there is a clear medical benefit in doing so (World Federation of Neurology, 1989; Bloch and Hayden, 1990). Testing a healthy child’s genetic status at the request of others removes their right to decide for themselves in adulthood (Harper and Clarke, 1990; Clarke, 1994).

In 1995, the American Society of Human Genetics (ASHG) and the American College of Medical Genetics (ACMG) published a joint report, which set out the current policy regarding genetic testing in adoption. They claimed that genetic information affected the ‘best interest of the child’, which now included ‘the child’s physical and psychological health, privacy interests and social development’ (1995, p. 761). They reasoned that the standard for testing adopted children should be equivalent to that of birth children and should not be used to ‘guarantee’ the child’s health prior to adoption. They also warned that ‘exaggerated assessments of historical risk’ may stigmatise the child and disadvantage their prospects for adoption (1995, p. 762).

Freundlich (1998) expressed similar concerns that adoption professionals and prospective adopters were advocating the use of predictive genetic testing for the purpose of ‘evaluating’ a child for adoption. Testing children prior to adoption may not only be harmful—potentially altering their status from adoptable to unadoptable—but implies a different standard of testing compared with children raised by biological parents. The principle of *equity* maintains that restrictions on seeking predictive genetic tests should apply equally to prospective adopters and biological parents. However, Jansen and Ross (2001) developed an interesting objection to the equity principle. They argued that parenting through adoption is not the same as parenting through birth. What they call the ‘matching argument’ holds that genetic information about children is relevant insofar as it may be used by adoption agencies to find a suitable family willing to adopt a child with a genetic disorder or, conversely, to rule out families who feel unable to parent such a child. They claim that restricting access to genetic testing may impede the state’s ability to find suitable adopters for children.

Although policy statements in the USA and the UK are generally consistent with respect to genetic testing of children, professional opinion appears divided on the issue of adoption. The recommendation of many professional groups in the USA is to restrict preadoption genetic testing for adult-onset disease. Even the most recent position statement of the ASHG, which acknowledges the matching argument, upholds its previous recommendation ‘that children awaiting adoption and adopted children be given the same consideration in genetic testing as children living with their biological parents’ (2015, p. 14). Professional policy in Europe remains in line with that of the ASHG. Recognising the importance of matching, the advice of the British Society for Human Genetics reminds practitioners that discussion could still be helpful even without testing, recommending that professionals have ‘an open discussion with the prospective parents’ to place the risks for the child in context (2010, p. 12).

Despite these restrictions, there are strong reasons why adoption agencies and prospective adopters may pursue genetic testing. Court decisions in the USA and UK have followed a pattern of imposing higher standards of care on adoption agencies to provide complete medical information (McDonald-Nunemaker, 1994). In 2003, the High Court for England and Wales judged that a local authority fell below the reasonable standard of care when it failed to disclose information about a child’s risk of behavioural difficulties to the adoptive parents (Hill *et al.*, 2010). With increasing pressure to disclose medical information, adoption agencies may perceive genetic testing as a tool for risk prediction. So too, prospective adopters may see genetic testing as an opportunity to reduce uncertainties about a child’s future health (Palmer, 2009). Whilst some argue for the expansion of preadoption genetic testing on the basis that it better serves children with special needs (Taylor, 2008; Taylor *et al.*, 2010), others argue that this will only introduce new uncertainties for adopters (Newson and Leonard, 2010; Leighton, 2014; Parker *et al.*, 2016; Newson *et al.*, 2016). We examine how the so-called matching argument in adoption copes with higher rates of uncertainty in ‘genome-wide’ testing.

## From genetic to genome-wide testing

Traditionally, genetic testing refers to relatively targeted investigations involving single-gene mutations or structural chromosome rearrangements, whereas ‘genome-wide’ testing can identify multiple variants in the DNA sequence across the entire human genome. Recent advances in genome-wide testing have increased the ability to detect genetic variation, thereby improving diagnosis for many conditions (Miller *et al.*, 2010). However, advances in detecting genetic variation are offset by the burden of interpretation. Many novel findings may be poorly understood and may not be causally related to the suspected condition. For these reasons, genome-wide testing is likely to pose new challenges for adoption.

The first line of investigation for a possible genetic cause of developmental delay is microarray-based comparative genomic hybridisation. But, in many cases, diagnostic testing may still fail to identify a genetic cause thereby challenging the widely held assumption that genetic testing is definitive and straightforward (Parker *et al.*, 2016). In fact, the outcomes of genome-wide testing can vary from those that are relevant to an investigation (i.e. finding or not finding an abnormality for a clinical problem), through those that may be relevant to future health (secondary and incidental findings) to those that are likely to sow confusion (i.e. findings of uncertain significance).

Microarray testing has improved diagnostic yield by identifying deletions and duplications of chromosomal material. Although some of these variants are well characterised (e.g. 22q11.2 deletion syndrome), the clinical significance of many other variants is not well understood. For instance, a microdeletion within 15q11.2 is commonly associated with a wide range of features including autistic traits and learning difficulties (Burnside *et al.*, 2011). However, variable expressivity and incomplete penetrance in populations suggests that this microdeletion is probably a risk factor rather than a causal explanation. This has raised concerns amongst medical practitioners that its detection amongst looked after children can be misleading and potentially stigmatising (Hamilton *et al.*, 2015).

With the mainstreaming of genomic medicine, adoption professionals will need to understand the relevance and complexity of biomedical information (Arribas-Ayllon *et al.*, 2020). Indeed, Jackson and Burke (2019) found that medical advisors and social workers in Wales reported many challenges concerning the appropriate use of genetic information in adoption and expressed the need for more training and support. However, these professionals believed that the impact of diagnosis on adoption was minimal. Another study in the USA explored social workers’ views of preadoption genetic testing (Erwin *et al.*, 2018), many of whom believed that testing was a useful ‘tool’ for adoption. But these participants had limited experience of genetic testing and assumed a model of predictive testing for single-gene conditions.

In the era of genomic medicine, some may believe that genetic testing removes uncertainty about a child’s future health, whilst others may need to surpass their negative associations of genetics to promote the best interests of the child (Taylor, 2008, 2010). Unlike previous studies that have invited social workers to speculate on their knowledge of genetic testing, this study infers knowledge and understanding from reported cases. We ask how do social workers and medical advisors retrospectively account for cases involving preadoption genetic testing?

## Method

This was a qualitative study funded by the Wellcome Trust (205644/Z/16/Z) to conduct preliminary research on the impact of genome-wide testing on adoption. Social workers were contacted through the National Adoption Service, all of whom were self-selecting as having first-hand experience of cases involving genetic testing. Medical advisors working in each of the local authorities were contacted individually via e-mail. Snowball sampling was used to contact participants who were singled out as having relevant experience; this widened our recruitment to England. Twenty participants were recruited through purposive sampling of social workers (*n* = 8), social work managers (*n* = 4) and medical advisors/community paediatricians (*n* = 8) working in adoption services in Wales. Medical advisors were all mid-to-late career, social workers were more diverse, ranging from the newly qualified to those with over thirty-five years’ experience.

Semi-structured qualitative interviews gathered detailed accounts of retrospective cases involving genetic testing in adoption. Treating research interviews as ‘accounts’ (Scott and Lyman, 1968) recognises that participants in interviews perform actions as well as representing states of affairs. Drawing on Austin’s (1961) classic formulation, there are two varieties of accounts, *justifications* and *excuses*, which are central to the management of responsibility. Accounts are therefore explanations or defences for conduct which may be heard as reasons for action (Antaki, 1994). These activities are especially relevant to professionals who construct reasoned accounts for genetic testing in adoption. Interviews explored a range of issues concerning: past cases of preadoption genetic testing, circumstances that trigger a genetic investigation, procedures for recording and communicating genetic information between professionals, practices of sharing information with prospective adopters, the impact of genetic information on post-placement experiences and whether a genetic diagnosis disadvantages a child’s prospects for adoption. All the interviews lasted between sixty and ninety minutes, were audio-recorded and subsequently transcribed verbatim.

Transcripts were coded via an iterative process of reading and noticing relevant phenomena, allowing the analyst (first author) to arrange data according to differences, commonalities and structures. Coded selections of data were compiled into a sub-corpus for group discussion (involving all three authors). Data extracts were selected to identify patterns of accounting and then arranged narratively around the central theme of ‘uncertainty’, which most participants recognised as a major issue in adoption. Each of the themes selected for analysis indicate how various forms of case construction, ethical deliberation and personal reflection are activities designed to justify professional conduct (see Supplementary Material for a statement on methodological limitations of the study). The study was approved by the School of Social Sciences Research Ethics Committee of Cardiff University (SREC/2229).

## Findings and analysis

A significant finding of the study was that participants discussed genetic testing in relation to historically higher levels of uncertainty in adoption. Social workers and medical advisors accounted for uncertainty in several ways: as a physical or future characteristic of the child, a lack of information about the child’s history, an unavoidable outcome of adoption and even a psychological reason for testing. The following themes reflect the range and variation of professional dilemmas including: *Imposition of testing* and *Juxtaposition of placement and the child’s autonomy*. We explain these themes and provide an analytic commentary on key extracts.

### Imposition of testing

All the medical advisors we interviewed reported cases involving inappropriate requests for testing prior to adoption. Whilst ‘clinical indication’ was cited as the most valid reason for testing a child, it was common to find this justification contrasted with the views of a ‘social worker’. We begin with a medical advisor giving an account of circumstances that trigger a genetic investigation:

Typically the testing is arranged when we have concerns about the child’s developmental progress, perhaps when a child presents with learning difficulties, and also when children have other neurodevelopmental conditions, perhaps diagnosis of autistic spectrum disorder. All of these factors would then initiate a screen to see, well is there a medical explanation? It would be based on those clinical indications, rather than whether a social worker thinks it’s a good idea to do a test. We do sometimes get pressured for that, in terms of when we know maybe there’s a wider family member who has a genetic change, sometimes the questions seem to be asked around, well, should we be testing this child who has perhaps a more distant [affected] relative, when they’re asymptomatic, maybe when they’re very young, for the same genetic change? I think that’s one of the headaches that array testing has thrown up.

In this account of ‘factors’ that typically warrant testing, clinical indication of neurodevelopmental problems is heard to be more legitimate than the social worker’s reasons for testing. Dilemmas arise when testing is requested for an ‘asymptomatic’ child because a ‘distant relative’ has a known mutation. The increasing accessibility and sensitivity of microarray testing is likely to create more of these dilemmas (‘headaches’) for medical professionals. Another medical advisor provided an even stronger account of inappropriate testing involving an asymptomatic child.

R: What if there is a family history but no dysmorphic features? […]

MA: Well I mean one of the ones I can think of is where there has been a family history of [inherited neuropathy] where there has been huge pressure from the [local] authority to undertake genetic testing and we are, it’s felt, and in discussion with genetics, it’s not appropriate to do so because the child [has] got no symptoms. There is nothing we are going to do about it, we aren’t going to treat anything at this point in time, and that the testing should be done at a later date if it is necessary. […]

R: Right, so why is that [pressure to test] then?

MA: Because uncertainty makes children more difficult to place […] I think they like to know that children don’t have things so that they can go forward with, this child has been tested and there is no evidence.

The medical advisor recalls a case of ‘huge pressure … to undertake genetic testing’. An account of *not* testing is justified on moral (‘it’s felt’) and clinical (‘no symptoms’) grounds with the support of clinical genetics. Asked to explain this tension, the medical advisor gives an emphatic reply: ‘Because uncertainty makes children more difficult to place’. This implies that local authorities believe that diagnostic testing will reduce uncertainty; it also suggests that prospective adopters are risk averse and therefore unlikely to adopt a child with a family history of genetic risk. This contradicts the findings of Jackson and Burke’s (2019, p. 270) study, where professionals reported that prospective parents ‘placed little importance on genetic testing’. Such views may indeed lead social workers to believe, like the participants in Erwin *et al.*’s (2018) study, that genetic testing is a useful ‘tool’ in adoption. But this assumes that a genetic test will show that ‘children don’t have things’ which overlooks the possibility that a child may indeed test positive.

When children ‘*do* have things’ like dysmorphic features and signs of developmental delay, other actors also appear to have a stake in testing. It is well known that Foetal Alcohol Spectrum Disorder (FASD) is overrepresented in the care system. Despite the guidance for clinical assessment, diagnosis can be difficult to establish in an individual child (Douzgou *et al.*, 2012). The current guidance (SIGN, 2019) recommends referral to a clinical geneticist if the features are atypical or prenatal exposure to alcohol is uncertain, but such referrals have often been used to *exclude other causes*. Below, a medical advisor describes a case in which diagnostic testing was ‘requested’ by the courts to confirm a diagnosis of FASD:

R: Are there any situations where you think CGH just shouldn’t be used?

MA: Foetal Alcohol Syndrome, I think if the case is strong history of using alcohol heavily during pregnancy, if the child is showing from birth some indication that he or she would be Foetal Alcohol Syndrome and if it proves by dysmorphism and behaviour why should I do an array CGH?

R: […] Who demands it? Where is the pressure coming from to have CGH in foetal alcohol?

MA: There is no pressure but it is mentioned in all the literature so it can be requested but shouldn’t be requested.

R: So sometimes the courts make you do stuff that you think isn’t medically- ?

MA: Ah, absolutely it happened to me. It did happen to me a child who is typical, classical foetal alcohol syndrome in every aspect and I have done my medical report and I said, this child is foetal alcohol syndrome around everything. And later on the court, without consulting me, asked for another community paediatrician who is interested in foetal alcohol syndrome to come and see this child from somewhere in [North England] and not even letting me know. And this person came and said, yes this child is foetal alcohol syndrome […] so the court requested for me to do an array CGH.

If the child meets the clinical criteria of FASD, the rhetorical question ‘why should I do an array CGH?’ seems to defy expectations that the child *should* be tested. The medical advisor denies the suggestion of external ‘pressure’, explaining that the guidelines condone testing; instead, her concern is oriented to *unnecessary* testing. A question about the involvement of ‘the courts’ elicits a case where the courts had intervened in the assessment of a child diagnosed with ‘classical’ FASD features. The medical advisor complains that the courts had surreptitiously approached ‘another … paediatrician’ who commissioned an array CGH. This case highlights different thresholds for testing amongst paediatricians as well as the motivations of the family courts to pursue testing. Later in the interview, she explained that ‘the child was going for adoption’ and the family courts wanted ‘proof’. In fact, several medical advisors reported that ‘social workers’, ‘special guardians’ and ‘the courts’ believed that a genetic test *for* FASD actually existed but, as one medical advisor put it, ‘there is no clinical investigation that’s going to give us that answer’.

Here, medical professionals are reporting the unwelcome ‘pressure’ to test children prior to adoption. Professional groups and authorities are misaligned in their roles and expectations of managing and reducing risk, whilst medical professionals are positioned as defending guidelines and their own clinical judgement.

### Juxtaposition of placement and the child’s autonomy

When social workers and medical professionals gave reasoned accounts of testing or not testing children, they did so by juxtaposing the practical ethics of placement with the principle-based ethics of the child’s autonomy. This is characteristic of the debates in the literature where it is claimed that rigorous adherence to the principle of autonomy can limit the child’s prospects of adoption (see Taylor, 2008). Proponents of the matching argument claim that the ‘exceptionalism’ of adoption is justification for testing (Jansen and Ross, 2001). Our focus is to show how the validity of these reasons rest on certain assumptions about genetic testing.

The following account comes from an experienced social worker who wanted to express her explicit disagreement with the guidelines. She presents the case of a ‘young child’ for whom testing was declined because he was too young to give consent. She claims that this decision made her role as ‘family finder’ difficult: ‘it ruled out 80% of the people that I was looking at and we had great, great difficulty finding adopters for him’. The issue, she explains, is the amount of uncertainty adopters are willing to tolerate:

Ultimately adoption is all about uncertainty and most adopters will go to the middle range of uncertainty, perhaps unknown father, some medical history in the family, perhaps some depression or personality disorder. And here we have a lot of the- those parents affected by drugs and alcohol. So that to me is a general scenario of our children’s background. You throw in something like a major illness and Huntington’s chorea is the other situation. That is too much, you know they will say no no. We have a system of matching children, we have a register- which is nationwide to try and link up families and children and we would get no, no, no. Very occasionally we’ve had a yes and we have placed a little boy with- I think it was neurofibromatosis with a family, but they are really exceptional. So for me, it closes down the pool of potential people and adoption I know is all about uncertainty and some people don’t want to know. Adults don’t want to know whether they have it or not. I suppose (laughs) I just think that it’s the right of the child to have knowledge about what we can say about them because there’s so much we can’t say. And lots of children don’t know who their fathers are so 50% of their make-up, we haven’t got a clue. So I’m sort of very for testing (laughs) […] Just the exact opposite of what’s in your guidelines, isn’t it?

We are told that ‘most adopters’ will accept an intermediate level of risk but only the ‘exceptional’ family will accept a child with a genetic disorder, an observation apparently confirmed by the adoption register. Not testing children for known risks ‘closes down the pool of potential people’, which implies that testing reduces uncertainty and therefore improves the child’s prospects of placement.

When social workers were presented with the autonomy argument, no one disputed its validity as a reason for not testing a child. But there was a clear distinction between medical professionals emphasising the *child’s* right to decide and social workers appealing to the responsibilities of *adults* to decide. In the following account, the manager of an adoption agency justifies adult decisions in terms of advocating the ‘best interests’ of the child:

I: …we’re talking about how the prospective parents are going to deal with this information, but there’s the whole ethical rights of the child. They don’t have a choice in whether this test is done.

SW: Absolutely. […] we’re always talking about the child being the centre of the process, aren’t we? So are we making those decisions for us as adults or for them as a child? You could argue that by knowing, we are custodians of this child, we have to make the decisions they can't make at that time in their life. And we are able to justify that by having that information means that we might be able to advocate for particular treatment in the future. Or we might be able to get to a point where we can explain the uncertainty in a manageable way to our child as they grow up. But yeah, it always has to come back to the child, and what is in their best interests. But you have got numerous professionals who are all very experienced and who are all very competent, and absolutely feel in their head that they have the best interest of that child, but they’re going to have conflicting agendas.

The manager is responding to the charge that the ‘whole ethical rights of the child’ may be ignored in adult decisions to test. In explaining that adoption services are entirely child-centred, she recasts the problem in terms of *who the test is for*. An argument in favour of *adults wanting to know* is justified in terms of custodial authority. Furthermore, having ‘that [genetic] information’ may have clinical utility ‘in the future’ where adults can ‘explain the uncertainty’ to the child over time. But we must assume that the manager is thinking of a case in which information *may* impact on the child’s clinical management *in the future*. This contradicts the medical advisor’s point above that testing should be deferred ‘if it’s not going to alter the child’s medical management’ *in the present*. The manager respectfully represents these differing professional views as ‘conflicting’ but valid interpretations of the best interests of the child.

Only one social worker in our study was able to explain the differences between predictive genetic testing and genome-wide testing. With over 30 years’ experience, she articulated the ethical dilemmas of placing a child with a family history of Huntington’s disease. Not testing the child prior to adoption was justified on the grounds that it ‘takes away’ the child’s right to choose for themselves in the future. This was immediately contrasted by a case involving a child who was tested for a suspected chromosome disorder. The following case illustrates how the outcomes of microarray testing are anything but definitive and straightforward:

I've had involvement with people whereby a child is deemed to have a chromosome disorder and I've been with some of my adopters to see a genetic counsellor. It's very confusing for people, I think, because they're often told, we know there's something different, but we don't know what the implications of that difference is. We will have to wait and see if we've got another child with the same chromosome disorder to find out what common difficulties might arise from that disorder. So adoptive parents are always aware that there’s a huge degree of uncertainty in terms of the background of their children, but the increases of information [are] helpful, to a degree. But it also throws up a lot of issues as well.

Even under the exemplary circumstances of accompanying adopters to see a ‘genetic counsellor’, the experience is described as ‘very confusing for people’. Adopting the voice of the counsellor, the social worker explains how the detection of ‘something different’ is the basis of uncertainty. She also gives an accurate account of how understanding chromosomal variants relies on observational studies of children who have ‘the same chromosome disorder’. Clinical studies look for correlations between poorly understood variants and their manifestation in children over time. Whilst genome-wide testing may reflect ‘increases of information’, they can also confirm the ‘huge degree of uncertainty’ that characterises the child’s background.

So how did the counsellor’s account of uncertainty affect the adoption? The social worker continues:

I think in [that] instance where we saw a genetic counsellor, because he was able to say we don't know what this abnormality is likely to give rise to, then it did cause my adopters to have second thoughts and not to proceed with the placement, which was sad. I felt bad for the child, really, but then if they weren't going to accept him come what may, then perhaps they weren't the right people for him anyhow. So it depends on what the circumstances are, because I suppose some things can give you a clearer answer than others because it's such an unfolding field, really, isn't it?

When children present with problems, and adults exercise their rights and responsibilities to know, the outcome of a genetic investigation does not necessarily resolve uncertainty. In the case above, uncertainty has simply acquired a new medical category. In line with the kind of risk aversion that seems to characterise contemporary adoption, we are told that the adopters had ‘second thoughts’ and the placement did not proceed. Perhaps, the most striking feature of the social worker’s account is her sympathy for the child who had not only lost a potential family but had now acquired the stigma of *genomic uncertainty*. It was common for social workers in our interviews to justify the breakdown of placement in terms of the character of adopters—that in the face of genetic risk perhaps they were not the ‘right people’ for the child after all.

In this theme, we hear the contrasting moral justifications of professionals arguing for and against testing. Social workers acknowledge the child’s autonomy but argue that the consensus position is too restrictive—adults have a responsibility as custodians to remove uncertainty as an impediment to adoption. Medical professionals seek to limit the right of adults by foregrounding the uncertainty of testing as well as the rights of children to choose for themselves in the future.

## Conclusion

With the mainstreaming of genomic medicine, genome-wide testing is likely to become a common line of investigation for diagnosing a whole variety of children presenting with neurodevelopmental problems. For these reasons, ethical debates over whether to test a child prior to adoption are likely to become more relevant to different professional groups, each of whom are charged with promoting the ‘best interests’ of the child.

Unlike previous studies that measure social workers’ hypothetical knowledge of preadoption genetic testing (Taylor *et al.*, 2010; Erwin *et al.*, 2018), this study explored actual cases accounted for by social workers and medical advisors. We have shown that conflict between professional groups occurred when two kinds of ‘exceptionalism’ were claimed. On the one side, medical professionals claimed that genetic information was exceptional because it contains unique data about individuals. Arguments in favour of autonomy claimed that genetic information belongs exclusively to individuals, and that their right to decide how and when this is used ought to be protected. On the other side, social workers claimed that adoption itself was exceptional and that access to genetic information may improve the child’s prospects for adoption. The so-called matching argument holds that the ‘right to know’ is a legitimate custodial responsibility of adults to place the child in a suitable home. We believe that both arguments are valid. Given that looked-after children are a *complex* paediatric population, it makes no sense to treat children eligible for adoption as the same *in principle* as those who are raised by their biological parents. In our study, social workers and medical professionals were united in recognising that adopted children encompass higher levels of uncertainty.

Our concern is not whether the ‘right to know’ in social care is unreasonable but whether the matching argument is realistic about what constitutes ‘genetic testing’. Like so many arguments that have informed genetics policy, those in favour of testing children are derived from scenarios involving *predictive genetic testing for Huntington’s Disease*, where outcomes are, for the most part, definite and clear. But genome-wide testing is not definite and clear—it can produce outcomes about small variations in chromosomal material of which very little are currently known. This has important implications for adoption because many social workers may be unaware of the potential *indeterminacy* of genome-wide testing. Indeed, if prospective adopters are as risk averse as we are led to believe, then receiving an unclear result (where the future implications of a child’s health and development are unknown) may give adopters further reason to withdraw from adoption. Also, if there is a prevailing assumption in social care that genetic testing is a tool to ‘guarantee’ a child’s future health, then it is incumbent upon social scientists to challenge this view. Uncertainty is likely to be an inherent aspect of the genomic investigation of children, which will require new ways of tolerating and communicating uncertainty (Newson *et al.*, 2016).

There is also a need to clarify exactly what role genetic testing plays in statutory health assessments for adoption. Interviews with medical professionals and social workers revealed contrasting views with respect to their role in diagnosis and clinical judgement. For medical professionals, a genetic investigation was not a proxy for clinical examination—genetic testing played a *contributory* role in clinical judgement, which may not by itself explain a child’s condition. However, some outside the medical profession may perceive testing as playing a *decisive* role in assessment, capable of superseding the clinical judgement of the medical practitioner. A case in point is the increasing pressure to use diagnostic testing for children with suspected FASD which, in a previous paper, we have shown can be mistakenly perceived as a test *for* FASD (Arribas-Ayllon *et al.*, 2020).

By drawing out these tensions amongst professionals, our aim is not to imply a deficit of understanding amongst social workers but to show that asymmetries of knowledge are a feature of different institutional values and competing priorities. Where genetic testing becomes entangled in the institutional politics of risk management, we find a strong desire to remove uncertainty from adoption. The hope that testing can be used to place children with parents who will be able to meet their needs is not an unreasonable goal but, as we have seen, even our most ‘advanced’ technologies are incapable of removing doubt from adoption. We believe that education should proceed through greater opportunities of knowledge-sharing amongst experienced social workers and through interdisciplinary cooperation with medical professionals. Education is necessary to provide competent counselling for prospective adopters to accept and tolerate uncertainty and to reduce pressure on local authorities to pursue genetic testing prior to adoption.

## Funding

This work was supported by the Wellcome Trust, grant number 205644/Z/16/Z.

## Supplementary material

Supplementary material is available at *British Journal of Social Work Journal* online.

## Supplementary Material

bcab017_Supplementary_Data
