# Supplementary material for: Can Genomics Remove Uncertainty from Adoption? Social Workers’ and Medical Advisors’ Accounts of Genetic Testing
Source: Br J Soc Work. 2021 Feb 24;52(2):719–37. doi: 10.1093/bjsw/bcab017 (PMC8929926; doi:10.1093/bjsw/bcab017)
Supplement: bcab017_Supplementary_Data [file bcab017_supplementary_data.docx]

**Methodological limitations**

This research makes several claims about professionals’ accounts of genetic testing in adoption. An obvious limitation of the study is the small number of participants. Are the findings then generalizable? If generalizability is defined as *statistical* and *probabilistic*, then the study is limited in its representativeness. However, if generalizability is interpreted ‘naturalistically’ (Smith 2018) to mean whether findings are *familiar* to or resonate with the reader, then the study is indeed representative of professionals’ experiences. In order to display naturalistic generalizability, it is necessary to provide enough detail of participants’ experiences. This is why qualitative studies of this kind generally show long interview extracts: to convey generalizability to its readers.

Another potential limitation of the study is the issue of research bias. There may be a *selection* or *confirmation bias* of singling out cases to identify controversies in genetic testing, however, the study takes the deliberate stance of identifying a range of patterns that indicate conflict between professionals. Other researchers may tell a different story. There is also the *self-report bias* of professionals who seek to give a good account of themselves or volunteer a specific point of view. But perhaps this is a strength of the study. For instance, one social worker volunteered in order to articulate her disagreement with the ethical guidelines on testing. Despite this motivation, her perspective helped to highlight tensions that exist in adoption.

No doubt this study favours the perspective of professionals. Future research ought to include those that make up the adoption triad, including the perspective of adopters and children. Post-adoption studies could track families where genetic testing had been referred or previously carried out to understand how families make sense of the implications of genetic and genomic information.

**Reference**

Smith, B. (2018) ‘Generalizability in qualitative research: misunderstandings, opportunities and recommendations for the sport and exercise sciences’, *Qualitative Research in Sport, Exercise and Health*, 10(1), pp.137-149.
